# A proteomic investigation of *Fusobacterium nucleatum* alkaline-induced biofilms

**DOI:** 10.1186/1471-2180-12-189

**Published:** 2012-09-03

**Authors:** Jactty Chew, Peter S Zilm, Janet M Fuss, Neville J Gully

**Affiliations:** 1 Oral Microbiology Laboratory, The School of Dentistry, The University of Adelaide, Adelaide 5005, Australia

**Keywords:** Fusobacterium nucleatum, Biofilms, Alkaline pH, Periodontal diseases, Proteomics

## Abstract

**Background:**

The Gram negative anaerobe *Fusobacterium nucleatum* has been implicated in the aetiology of periodontal diseases. Although frequently isolated from healthy dental plaque, its numbers and proportion increase in plaque associated with disease. One of the significant physico-chemical changes in the diseased gingival sulcus is increased environmental pH. When grown under controlled conditions in our laboratory, *F. nucleatum* subspecies *polymorphum* formed mono-culture biofilms when cultured at pH 8.2. Biofilm formation is a survival strategy for bacteria, often associated with altered physiology and increased virulence. A proteomic approach was used to understand the phenotypic changes in *F. nucleatum* cells associated with alkaline induced biofilms. The proteomic based identification of significantly altered proteins was verified where possible using additional methods including quantitative real-time PCR (qRT-PCR), enzyme assay, acidic end-product analysis, intracellular polyglucose assay and Western blotting.

**Results:**

Of 421 proteins detected on two-dimensional electrophoresis gels, spot densities of 54 proteins varied significantly (*p* < 0.05) in *F. nucleatum* cultured at pH 8.2 compared to growth at pH 7.4. Proteins that were differentially produced in biofilm cells were associated with the functional classes; metabolic enzymes, transport, stress response and hypothetical proteins. Our results suggest that biofilm cells were more metabolically efficient than planktonic cells as changes to amino acid and glucose metabolism generated additional energy needed for survival in a sub-optimal environment. The intracellular concentration of stress response proteins including heat shock protein GroEL and recombinational protein RecA increased markedly in the alkaline environment. A significant finding was the increased abundance of an adhesin, Fusobacterial outer membrane protein A (FomA). This surface protein is known for its capacity to bind to a vast number of bacterial species and human epithelial cells and its increased abundance was associated with biofilm formation.

**Conclusion:**

This investigation identified a number of proteins that were significantly altered by *F. nucleatum* in response to alkaline conditions similar to those reported in diseased periodontal pockets. The results provide insight into the adaptive mechanisms used by *F. nucleatum* biofilms in response to pH increase in the host environment.

## Background

Bacteria in nature are exposed to changing environmental conditions; they sense and detect signals from their surroundings and gene expression is regulated in response to specific cues in harsh environments to adapt and survive [1]. The anaerobic Gram negative oral bacterium, *Fusobacterium nucleatum*, is frequently isolated from both supra- and sub-gingival dental plaque in humans and has been implicated in the aetiology of periodontal disease [2-4]. This bacterium is one of the most common oral species isolated from human extra-oral infections and abscesses including blood, brain, liver, abdomen and genital tract [5]. Increasing evidence also suggests that *F. nucleatum* is associated with an increased risk of preterm birth [5-8] while two latest studies indicated a possible association between the presence of *F. nucleatum* and bowel tumors [9,10].

Studies have reported that the pH of the periodontal pocket in humans suffering from periodontitis is alkaline and may be as high as 8.9 [11-13]. It is also reported that localised pH gradients ranging between 3 and 8 occur within a 10-species oral biofilm model [14]. The alkalinity in the disease state is largely due to the release of ammonium ions produced from the catabolism of amino acids and peptides derived from gingival crevicular fluid (GCF) by proteolytic bacteria [15,16]. Previous studies in our laboratory showed that when grown in a chemostat between pH 6 and 8, *F. nucleatum* grew as planktonic culture [17]. We have also reported that increasing the culture pH to 8.2 induced biofilm growth and the cells exhibited significant increases in length and surface hydrophobicity [18]. This pH alkaline-induced phenotypic switch to biofilm growth observed may be an adaptive mechanism in response to adverse environmental pH that occurs during the progression of periodontal disease *in vivo*. This bacterium has been demonstrated to survive in calcium hydroxide treated root canal systems at pH 9.0 [19] and in a separate study, biofilm growth conferred protection to root canal bacteria at pH 10 [20]. Biofilm formation by *F. nucleatum* may provide protection to cells when exposed to alkaline environments. Bacteria growing in biofilms exhibit altered phenotypes and are more resistant to antimicrobial agents and the host immune system [21]. The characterisation of biofilms has revealed that cells within them exhibit different concentrations in proteins involved in metabolism, transport and regulation [22-25]. Protein regulation in *F. nucleatum* in response to acidic (pH 6.4) and mild alkaline (pH 7.4 and 7.8) has been reported [26,27].

The present study uses a proteomic approach to examine changes in protein expression by *F. nucleatum* associated with biofilm formation induced by growth at pH 8.2. Where possible, the expression of proteins that was significantly altered was validated using enzyme assay, acidic end-product analysis, Western blotting and qRT-PCR. This study identified 54 proteins with significantly altered concentrations in alkaline-induced *F. nucleatum* biofilms that may reflect changes in cellular functions that occur in the diseased environment.

## Methods

### Bacterial culture conditions

*F. nucleatum* subsp. *polymorphum* (ATCC 10953) was purchased from Cryosite (NSW, Australia) and maintained on anaerobic blood agar plates (Thermo Fischer, Vic, Australia). The bacterium was cultured anaerobically using a model C-30 Bio-Flo Chemostat (New Brunswick Scientific, NJ, USA) as previously described, with minor modifications [26]. Briefly, a chemically defined growth medium based on that of van der Hoeven [28] was supplemented with 10 mM glucose, 20 mM glutamic acid, 10 mM histidine and 10 mM lysine (all other amino acids were 1 mM). Amino acids were purchased from Sigma Aldrich (St Louis, MO, USA). During planktonic growth, the medium was pumped at a flow rate of 27 mL/h to give an imposed dilution rate of D=0.069/h. Using the relationship, Tg (generation time)=ln 2/D, this gave a bacterial generation time of 10 h. Such generation time of the culture mimics the growth rate of bacteria in mature dental plaque (generation time between 7–12 h) [29]. Initially, the culture was maintained at pH 7.4 ± 0.1 which was optimal for growth of the organism at 37°C [17]. The planktonic culture was harvested after steady state was achieved (10 generations). The culture was removed from the culture vessel and stored at −80°C until use. The growth pH was then increased by 0.2 unit increments to 8.2 ± 0.1 over an 8 h period. Several hours after pH 8.2 was achieved, *F. nucleatum* cells adhered to surfaces of the culture vessel and formed biofilms. Biofilm cells were harvested by increasing culture agitation during sampling to dislodge adherent cells. Cell aggregates from detached biofilms were allowed to settle for 2 min. Planktonic cells were carefully decanted and the remaining biofilm cells were used for further analyses. Bacterial cultures grown under both pH conditions were harvested daily, for five consecutive days, and pooled as biological replicates.

### Sample preparation for proteomic analysis

Bacterial cells were collected by centrifugation (8,000 × *g*, 4°C, 10 min) and lysed by sonication (Soniprobe, Dawe Instruments, England; 1.8 A for 5 cycles, 10 s each) on ice. Unbroken cells were removed by centrifugation at 2,500 × *g* (4°C, 10 min). Centrifugation of cell free lysates at 20,000 × *g* (4°C, 30 min) was performed to pellet the cell envelope (inner and outer membranes). Cytoplasmic proteins present in the supernatant were prepared as described previously [26] and membrane proteins were prepared from the cell envelope fraction using the method described by Molloy and colleagues [30] with slight modifications.

Briefly, precipitation of cytoplasmic proteins was performed by incubating samples with ice cold acetone (four sample volumes) containing 50 mM dithiothreitol (DTT; Sigma Aldrich, St Louis, MO, USA) overnight at −20°C. Protein precipitate was collected by centrifugation at 10,000 × *g* (2°C, 30 min). Membrane proteins were extracted by resuspending cell pellets in sodium carbonate (0.1 M, pH 11) and stirred on ice for 1 h. The carbonate-treated membranes were collected by ultra-centrifugation (115,000 × *g*, 4°C, 1 h). Extracted cytoplasmic and membrane proteins were then solubilised with ReadyPrep Reagent 3 (Bio-Rad Laboratories, CA, USA) containing 5 M urea, 2 M thiourea, 2% (w/v) CHAPS, 2% (w/v) detergent sulfobetaine 3–10, 40 mM Tris, 0.2% Bio-lyte 3/10 and 2 mM tributyl phosphine and stored at −80°C until required.

### Protein separation by two-dimensional gel electrophoresis (2DE)

Protein quantification was performed using Reducing Agent and Detergent Compatible Protein Assay Kit (Bio-Rad Laboratories, CA, USA) prior to 2DE. Gel-based isoelectric focusing (IEF) was performed using a PROTEAN IEF Cell (Bio-Rad Laboratories, CA, USA) using pre-cast Immobilised pH Gradient (IPG) strips with an isoelectric point (pI) range of 4–7 or 7–10 and proteins were cup-loaded onto the anode end of IPG strips. Optimal protein load and IEF running conditions are listed in Additional file 1: Table S1. Cytoplasmic proteins with a pI between 7 and 10 required an additional liquid-based IEF separation prior to 2DE. A total of 10 mg of solubilised cytoplasmic proteins were separated into 10 fractions between pI 3 and 10 using a MicroRotofor Liquid-Phase IEF Cell (Bio-Rad Laboratories, CA, USA). Liquid-based IEF was performed at 20°C at 1 W for 2 h. The fractions between pI 7 and 10 were pooled and following protein determination, separated by 2DE.

Following 2DE IEF, IPG strips were incubated in 2% (w/v) DTT in equilibration buffer (6 M urea, 2% (w/v) SDS, 0.05 M Tris/HCl buffer (pH 8.8) and 20% (v/v) glycerol), followed by 2.5% (w/v) iodoacetamide in equilibration buffer for 15 min each. Proteins were then separated on 20 × 20 cm polyacrylamide (12% T, 3.3% C, 0.1% SDS, 375 mM Tris/HCl, pH 8.8) gels using a PROTEAN II XL Multi-Cell (Bio-Rad Laboratories, CA, USA) which allowed six gels to be run simultaneously. Gels were stained with either Coomassie Brilliant Blue R-250 (Sigma Aldrich, MO, USA) or Flamingo Fluorescent Stain (Bio-Rad Laboratories, CA, USA) and scanned using a GS-800 Densitometer (Bio-Rad Laboratories, CA, USA) or Typhoon Scanner (GE Healthcare, Buckinghamshire, UK), respectively.

### Image acquisition and analysis

Image analysis of the 2-DE gels was performed using PD-Quest 7.2 Software (Bio-Rad Laboratories, CA, USA). Six gels were produced for each pI range (4–7 and 7–10) for cytoplasmic and cell membrane proteins from either biofilm or planktonic cells (48 gels in total). Replicate groups containing four to six highly reproducible gels from either planktonic or biofilm cells were used for analysis. Spot intensities were normalised using the total density in gels. Spot detection was performed using the automated spot detection algorithm before being checked and matched manually. Matchsets containing gel images were created to identify proteins that showed significant changes in concentration (at least two-fold changes in spot intensities at a significance level of *p* <0.05, Student’s *t*-test). Analysis sets comparing growth conditions containing proteins that appeared in all replicate gels which showed significant quantitative changes were identified and proteins were excised from gels for MS analysis and protein identification.

### Matrix assisted laser deionisation mass spectrometry (MALDI-MS)

All mass spectrometry (MS) instruments and analysis software were purchased from Bruker Daltonics GmbH (Bremen, Germany). The excised protein spots were digested with trypsin, destained and digested as described before [27].

One microlitre of each sample was applied to a 600 μm AnchorChip according to the α-cyano-4-hydroxycinnamic acid method [31]. MALDI-TOF mass spectra were acquired using a Bruker Ultraflex III MALDI-TOF/TOF mass spectrometer operating in reflectron mode under the control of the flexControl software (Version 3.0). Peptide standards were used to perform external calibration under identical conditions. MS spectra were collected randomly across each AnchorChip spot. Optimal laser intensity and shot count were both operator determined. Those spectra which exhibited high signal to noise MS peaks were summed together to generate a final peptide MS fingerprint spectrum. Between three and six of the most highly abundant sample ions (i.e. non-trypsin and non-keratin) were selected as precursors for MS/MS analysis. MALDI-TOF/TOF was performed in the LIFT mode using the same spot on the target [32]. MS and MS/MS spectra were subjected to smoothing, background subtraction and peak detection using flexAnalysis (version 3.0). The spectra and mass lists were exported to BioTools (version 3.1). The MS and corresponding MS/MS spectra were combined and submitted to the in-house Mascot database-searching engine (version 2.2, Matrix Science: http://www.matrixscience.com) using the following specifications:

Taxanomy: Eubacteria

Database: NCBI non-redundant 20080622, 20081114 and 20100216

Fixed modifications: carbamidomethyl (C)

Variable modifications: oxidation (M)

Mass tol MS: 50 p.p.m

MS/MS tol: 0.5 Da

Missed cleavages: 1

Protein identification was based upon the MOWSE and probability scored generated by the software. Based on the combined MS/Ms data, samples that returned a positive ‘hit’ were submitted independently to Mascot.

### Liquid chromatography-ESI mass spectrometry (MS and MS/MS)

Samples that failed to give sufficient spectra using MALDI MS/MS for accurate protein identification were further analysed using LC-ESI ion trap MS/MS. Peptides were separated by chromatography using an Agilent Protein ID Chip column assembly (40 nL trap column with 0.075 × 43 mm C-18 analytical column) housed in an Agilent HPLC-Chip Cube Interface (Agilent Technologies, CA, USA) connected to an a HCT ultra 3D-Ion-Trap mass spectrometer. The column was equilibrated with 4% acetonitrile containing 0.1% formic acid at 0.5 μL min^-1^ and the samples eluted with an acetonitrile gradient (4%-31% in 32 min). MS/MS spectra of ionisable species were acquired in a data-dependant fashion as follows: Ionisable species (300 < m/z < 1200) were trapped and the two most intense ions in the scan were independently fragmented by collision-induced dissociation. Post acquisition, MS and MS/MS spectra were subjected to peak detection using Bruker’s DataAnalysis software (version 3.4). Data were imported into BioTools. MS/MS data were searched as described above, but with an MS mass tolerance and MS/MS tol of 0.3 and 0.4 Da, respectively, and a peptide charge of 1+, 2+ and 3 + .

### Western blotting analysis

The intracellular concentrations of heat shock protein (HSP) GroEL and a recombination protein RecA were analysed by Western blotting. Aliquots of cell lysates from both planktonic and biofilm cultures equivalent to 15 μg of protein, were separated by electrophoresis on 12%T 3.3% C polyacrylamide gels (100 V, 1.5 h) [33]. The proteins were then electro-transferred to an Immuno-Blot PVDF membrane (Bio-Rad Laboratories, CA, USA) using Mini Trans-Blot Cell (250 mA, 2 h) (Bio-Rad Laboratories, CA, USA) followed by blocking (1 h, room temperature) using 5% (w/v) ECL Blocking Agent (GE Healthcare, Buckinghamshire, UK). The washed membrane was then treated with either mouse anti-human Hsp60 monoclonal antibody (SPA-087, Stressgen Biotechnologies, British Columbia, Canada) diluted 1:1000 or mouse anti-*E. coli* RecA monoclonal antibody (MD-02 + 3, MBL International, IF, USA) diluted 1:1000 for 24 h at 4°C. The washed membrane was then probed for 1 h at room temperature with anti-mouse alkaline phosphatase conjugate secondary antibody (1 mAB: 5000 BSA- tris-buffered saline-tween 20 (TBS-T)). The target protein was detected using ECF substrate and scanned using a Typhoon Scanner. The expression of the protein was analysed using ImageQuant TL software. EFC substrate, Typhoon Scanner and ImageQuant TL software were purchased from GE Healthcare (Buckinghamshire, UK).

### Quantitative real-time PCR (qRTPCR)

Gene sequences of *groEL*, *dnaK* and *recA* and 16S rRNA were retrieved from the Oralgen Databases (http://www.oralgen.lanl.gov) and primers were designed using the web-based tool Primer 3-PCR (Additional file 2: Table S2). 16S rRNA was used as reference gene.

Bacterial samples from each culture type (4 mL) were harvested and incubated in 4 mL of RNA*later* (Ambion, Austin, TX, USA) overnight at 4°C. RNA*later* was then removed by centrifugation (5,000 × *g*, 4°C, 15 min). Cell pellets were resuspended in 1 mL of fresh RNA*later* and stored at −80°C until required. Total RNA was extracted from the bacterial pellets using the RiboPure-Bacteria Kit (Ambion, TX, USA) following the manufacturer’s instructions. Bacterial cDNA templates were generated from 1 μg RNA by reverse transcription using a SuperScript® Vilo^™^ cDNA Synthesis Kit (Invitrogen, CA, USA). qRT-PCR was performed using a Corbett Rotor-Gene RG-3000 Thermal Cycler (Qiagen, Hilden, Germany) using a standard curve method. Each PCR run consisted of a standard curve and five biological replicate samples for each growth pH. All standards and samples were performed in triplicate. The total reaction volume of 20 μL consisted of 2 μL of each forward and reverse primer, 10 μL of Platinum SYBR Green qPCR SuperMix-UDG (Taq DNA polymerase, SYBR Green I dye, Tris–HCl, KCl, 6 mM MgCl_2_, 400 μM dGTP, 400 μM dCTP, 800 μM dUT, UGG and stabilizers; Invitrogen, CA, USA), 5 μL dH_2_O and 1 μL of diluted cDNA. The conditions for amplification cycles were as follows: 40 cycles consisting of denaturation at 95°C for 15 s, annealing at 60°C for 60 s, and extension at 72°C for 30 s.

### NAD-specific glutamate dehydrogenase (GDH) assay

Planktonic and biofilm cells were harvested and lysed as described above. A protein assay was performed using Coomassie Plus Protein Assay Kit (Thermo Scientific, Rockford, IL, USA) on each lysate and an equal amount of cell protein was used to measure GDH activity based on the protocol proposed by Irwin and co-workers [34] with slight modifications. The amount of enzyme in samples was determined by measuring the rate of conversion of NAD^+^ to NADH over 5 min, a reaction that generates a proportional increase in absorbance at 340 nm and was measured spectrophotometrically (Lambda 5 Spectrophotometer, Perkin Elmers, Bodenseewerk, Germany). Reaction mixtures contained 1 mM NAD^+^, 4 mM L-glutamate, 50 mM sodium pyrophosphate buffer (pH 8.8) and 50 μL of cell lysate. GDH activity in cell lysates was expressed in GDH unit per mg of cell protein. GDH from bovine liver (Sigma Aldrich, MO, USA) was used to construct a standard curve.

### Metabolic end-product and intracellular polysaccharide (IP) analyses

Acidic end-product analysis was performed on an ion-exclusion HPLC (Waters, MA, USA) protocol based on that of Gully and Rogers [35]. IP concentrations were determined using the method of Hamilton and colleagues [36].

## Results and discussion

### Changes in protein expression induced by pH 8.2 in *F. nucleatum*

**Figure 1:**
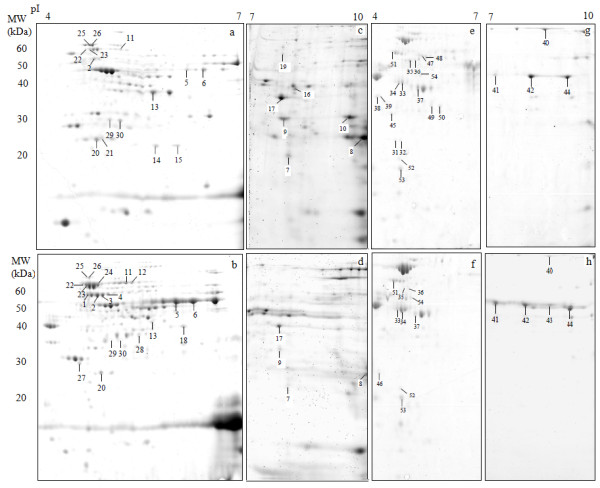
**Representative 2DE gel images of planktonic (pH 7.4; a, c, e and g) and biofilm cells (pH 8.2; b, d, f and h).** a - d cytoplasmic proteins; e - h cell envelope proteins. Proteins that were differentially produced are annotated. Refer to Table 1 for protein identification and abundance.

The genome of *F. nucleatum* subsp. *polymorphum* (ATCC 1953) codes for 2067 open reading frames (ORFs) [5]. In this study, we examined proteins that are within pI range 4–10, and molecular weight (MW) range 10 and 80 kDa, which represents approximately 80% of the *F. nucleatum* genome [26]. Previous studies resolved whole cell- or cytoplasmic-protein subsets within a 4–8 pI range [26,37-39]. We have also reported the expression of cell envelope proteins in *F. nucleatum* (pI 4–10) grown at pH 7.8 [27]. In comparison, the present study examined both cytoplasmic and cell membrane protein expression (pI range 4–10) following growth at pH 8.2. As proteins with basic pIs are considered to be difficult to resolve on 2DE gels [40], optimisation of 2DE conditions was performed to yield satisfactory protein resolution (Additional file 1: Table S1 and Figure 1). Without MicroRotofor-IEF separation, only a small number of cytoplasmic proteins between pI 7 and 10 were resolved on 2DE gels that contained excessive vertical streaking (data not shown). This was likely due to the comparatively high abundance of soluble proteins in the pI 4–7 range in samples. Prior to 2DE, therefore, proteins with a pI < 7 were removed. Protein assay of pooled fractions confirmed that the ratio of acidic (pI 4–7) to basic (pI 7–10) proteins was approximately 4:1 (data not shown). The overcrowding of acidic proteins (pI 4–7) has been reported in microbial species including the parasitic protozoa *Leishenia amazonensis*[41]. In this study, a reduced amount (100 μl) of sample containing enriched cytoplasmic proteins (pI 7–10) was loaded onto 11 cm IPG strips. Due to the reduced protein load, gels were stained with Flamingo Fluorescent stain (Additional file 1: Table S1). As only 30% of the bacterial genome encodes for membrane proteins, we also included the separation of cell envelope and cytoplasmic proteins prior to 2DE to improve the detection of membrane proteins [42].

A total of 31 gels were used for expression analysis. 421 proteins, representing 330 cytoplasmic and 91 membrane proteins, with a pI between 4 and 10 and a MW between 10 and 80 kDa were separated and visualised using Coomassie/Flamingo Fluorescent stains (Additional file 1: Table S1). Comparison of 2DE gels representing growth at pH 7.4 and 8.2 revealed that the intracellular concentrations of 54 proteins were significantly (p < 0.05) altered at least two-fold (Table 1). The abundance of 23 proteins either increased marked or exclusively detected in biofilm cells while 31 proteins either decreased in biofilm cells or were only detected in planktonic cells. A number of proteins were identified as potential isoforms arising from post-translational modifications indicated by altered pI and/or MW. Table 1 summarises proteins identified and groups them according to their functional classes.

**Table 1:** Significantly regulated protein expression in response to growth pH 8.2

**Function**	**Protein name**	**Accession number**^**1**^	**Gene ID**^**2**^	**Spot number**^**3**^	**Fraction**^**4**^	**%Seq MS/MS**^**5**^	**Density**^**6**^**(×10**^**3**^**)**		**Mean Ratio**^**7**^	***p*-value**^**8**^	**Pred. MW/pI**^**9**^	**Obs. MW/pI**^**10**^
							**pH 8.2**	**pH 7.4**				
Cellular energy												
2-oxoglutarate pathway	NAD-specific glutamate dehydrogenase (EC 1.4.1.2)	148324272	1750	5	C	29	18.5	3.9	4.8	0.01	46.6/6.1	48/6.2
				6	C	52	18.8	6.0	3.1	0.01		48/6.6
				7^	C	10	1.6	7.5	0.2	0.02		35/7.9
				8^	C	31	5.9	49.3	0.1	0.01		23/9.5
				9^	C	32	2.7	16.6	0.2	0.01		24/8.0
				10^	C	30	nd	24.4	-	0.01		28/9.0
	Gluaconyl-CoA decarboxylase A subunit (EC 4.1.1.70)	148322789	0224	11	C	40	2.5	1.1	2.3	0.02	64.1/5.1	62/5.3
				12	C	34	1.7	nd	+	0.02		62/5.4
	Glutamate formiminotransferase (EC 2.1.2.5)	148323936	1404	13	C	47	0.6	14.3	0.1	0.01	36.0/5.5	38/5.6
Butanoate synthesis	Butanoate: acetoacetate CoA transferase α subunit (EC 2.8.3.9)	148323516	0970	14^	C	36	nd	3.7	-	0.01	23.3/6.1	23/5.8
				15^	C	50	nd	2.9	-	0.01		23/6.1
	Butyryl-CoA dehydrogenase (EC 1.3.99.2)	148323999	1467	16^	C	31	nd	6.7	-	0.05	41.8/7.8	39/8.1
Acetate synthesis	Phosphate acetyltransferase (EC 2.3.1.8)	148323174	0618	17^	C	7	3.8	nd	+	0.05	36.0/7.6	39/7.6
Pyruvate metabolism	D-lactate dehydrogenase (EC 1.1.1.28)	148324271	1749	18	C	41	1.2	nd	+	0.05	37.8/6.1	36/6.1
	Pyruvate synthase (EC 1.2.7.1)	148324582	2072	19^	C	1	nd	1.3	-	0.05	132.1/6.7	58/7.7
One carbon pool by folate	Methenyltetrahydrofolate cyclohydrolase (EC 3.5.4.9)	148323933	1401	31	M	28	nd	2.0	-	0.01	22.9/4.9	19/4.9
				32	M	12	nd	3.3	-	0.01		19/5.0
Transport												
Substrate transport	Di-peptide binding protein DppA	148323000	0440	1	C	8	1.6	nd	+	0.02	56.9/5.3	55/4.6
				2	C	6	5.9	0.7	8.6	0.02		55/4.8
				3	C	5	4.1	nd	+	0.02		55/4.9
				4	C	5	1.8	nd	+	0.02		55/5.0
	Dicarboxylate: Proton (H^+^) TRAP-T (tripartite ATP-independent periplasmic) family transporter binding protein	148323082	0524	33	M	10	100.1	1.7	6	0.01	28.9/5.0	39/4.9
				34	M	13	57.1	0.6	10	0.02		39/5.0
	RND (resistance-nodulation-cell division) superfamily antiporter	148323066	0508	35	M	10	1.0	3.9	0.3	0.01	40.8/5.2	43/5.1
				36		7	1.3	3.2	0.4	0.05		43/5.2
	TTT (tripartite tricarboxylate transporter) family receptor protein	148322550	2414	37	M	21	1.3	3.2	0.1	0.04	35.2/5.5	33/5.2
	ABC (ATP binding cassette) superfamily transporter binding protein	148322870	0306	38	M	24	1.1	nd	-	0.01	32.0/4.7	32/4.6
				39	M	24	1.3	nd	-	0.01		32/4.6
Porin	OmpIP family outer membrane porin	148322338	2196	40	M	8	10.6	27.9	0.4	0.02	78.1/8.8	75/8.8
	Fusobacterial outer membrane protein A (FomA)	148323518	0972	41	M	12	63.6	14.3	4.4	0.03	42.3/8.4	42/7.8
				42	M	12	58.1	2.3	25.8	0.03		42/8.1
				43	M	14	18.3	nd	+	0.01		42/8.6
				44	M	5	23.3	1.6	7.7	0.01		40/9.2
Electron acceptor	Electron transfer flavoprotein subunit A	148324001	1469	20	C	9	0.1	3.2	0.0	0.01	42.5/5.5	25/5.2
				21	C	19	nd	1.1	-	0.01		25/5.4
	Electron transfer flavoprotein subunit B	148324000	1468	45	M	15	nd	5.1	-	0.01	28.6/4.7	27/4.7
	NADH dehydrogenase (ubiquinones), RnfG subunit	148322329	2186	46	M	10	0.9	nd	+	0.05	19.0/4.6	18/4.6
Stress response												
Heat shock proteins (HSP)	60 kDa chaperonin (GroEL)	29839341	1329	22	C	*	0.9	0.3	3.2	0.05	57.5/5.0	57/4.7
				23	C	*	3.9	0.8	4.9	0.01		57/4.7
				24	C	*	3.8	nd	+	0.05		57/4.9
	70 kDa chaperone protein (DnaK)	40643393	1258	25	C	*	0.7	3.2	0.2	0.01	65.3/5.0	65/4.7
				26	C	*	0.2	2.5	0.1	0.05		65/4.7
	Peptidyl-prolyl cis-trans isomerase	148322857	0293	27	C	55	0.8	nd	+	0.01	26.7/5.0	27/4.6
DNA repair	Recombination protein RecA	148324333	1811	28	C	59	3.4	nd	+	0.05	35.2/5.6	35/5.5
Protein synthesis												
Translation	Elongation factor EF-Ts	148323585	1043	29	C	*	0.2	2.0	0.1	0.02	33.0/5.3	35/5.1
				30	C	*	0.7	2.8	0.1	0.03		35/5.3
				54	M	29	nd	2.6	-			38/5.2
	Elongation factor EF-Tu	148322297	2153	47	M	9	nd	5.5	-	0.01	43.4/5.1	45/5.5
				48	M	10	nd	6.2	-	0.01		45/5.6
	Ribosomal protein S2	148323584	1042	49	M	9	nd	3.0	-	0.01	27.9/5.3	30/5.5
				50	M	13	nd	3.2	-	0.01		29/5.7
Hypothetical protein	Hypothetical protein FNP_1008	148323554	1008	51	M	6	20.0	6.6	3.0	0.01	45.5/4.9	45/4.9
	Hypothetical protein FNP_0594	148323151	0594	52	M	12	0.8	2.9	0.3	0.04	9.9/4.7	11/5.2
	Hypothetical protein FNP_0283	148322501	0238	53	M	6	6.6	16.6	0.4	0.01	18.0/5.0	10/5.0

All proteins were identified using MALDI MS/MS except those marked with ‘^’ were identified using LC-ESI MS/MS.

^1^Protein accession number on National Centre for Biotechnology Information (NCBI).

^2^Annotated gene ID on Oralgen Database (http://www.oralgen.lanl.gov/_index.html).

^3^Spot number as shown in Figure 1.

^4^Protein present in either cytoplasmic (C) or membrane (M) fraction.

^5^Percentage of sequenced peptides from MS/MS analysis found to match the identified protein.

^6^The average protein density of biofilm cells (pH 8.2) compared to planktonic cells (pH 7.4) on gel images determined by PD-Quest software V. 7.2.

^7^Mean ratio of biofilm cell protein quantity against planktonic cell protein quantity; calculation based on 3–5 replicate gels.

^8^*p*-value^,^ Student *t*-test.

^9^Predicted molecular weight (MW) and isoelectric point (pI) of protein determined from Oralgen Databases.

^10^Observed MW and pI of protein determined from 2DE gels (Figure 1).

^+^Proteins that were only resolved in biofilm cells.

^-^Proteins that were only resolved in planktonic cells.

nd – not detected on 2DE gels.

Earlier studies in our laboratory showed that the regulation of proteins associated with energy production, transport and protein folding occurred in planktonic cells cultured at pH 7.8 [26,27]. While the present study reports a similar change in protein expression patterns at pH 8.2, we have identified 32 proteins that are altered in response to growth at pH 8.2. It is likely that these proteins may be associated with the altered morphology and biofilm formation observed at the higher pH.

### Changes in cellular metabolism

**Figure 2:**
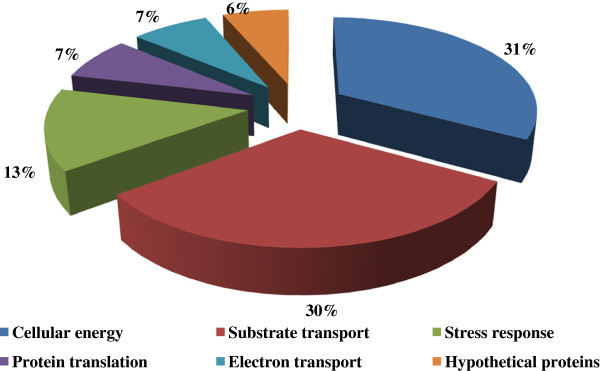
Representation of protein groups that were regulated at pH 8.2 compared to 7.4.

Our data show that metabolic enzyme production was closely associated with a change to biofilm growth at pH 8.2. 31% (17 proteins) of all identified proteins were associated with metabolism and 30% (16 proteins) were substrate-transporters (Table 1 and Figure 2). *F. nucleatum* is able to catabolise both sugars and amino acids as energy sources [17,19,43], in contrast to the periodontal pathogens *Porphyromonas gingivalis*[20] and *Treponema denticola*[44]. Histidine and glutamic acid are among the amino acids that are readily metabolised by *F. nucleatum* to generate energy and produce ammonia, acetate and butanoate as end-products [45-47]. The bacterium also ferments sugars (glucose, galactose and fructose) to produce a mixture of acetate, formate and lactate [48].

**Figure 3:**
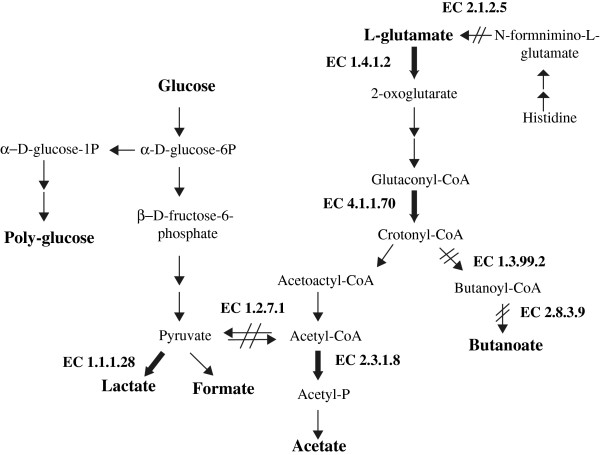
**Pathways for glucose and histidine/glutamate catabolism in *F. nucleatum. *** Significantly regulated enzymes detected in this study at pH 8.2 are indicated by the enzyme commission (E.C) numbers (Refer to Table 1). Bold arrows indicate increased enzyme levels while double-slash indicates decreased enzyme expression.

In the present study, key enzymes involved in the catabolism of glutamate and histidine via the 2-oxoglutarate pathway and pyruvate were significantly altered in biofilm cells (Table 1). A previous study of *F. nucleatum* cultured at pH 6.4, 7.4 and 7.8 also revealed the regulation of metabolic enzymes [26]. In contrast to this finding, we found that no glycolytic enzyme concentrations were altered in biofilm cells grown at pH 8.2 compared to planktonic cells grown at 7.4. However, a three-fold increase in glucose utilisation and IP was observed (Table 2, Figure 3). It is possible that the observed increase in glucose storage may play an important role in the organism’s survival during periods of nutrient limitation when exposed to pH 8.2 [43,49,50]. Although the expression of glycolytic enzymes was not significantly altered, an increase in lactate dehydrogenase (LDH) (EC 1.1.2.8) and a three-fold increase in lactate production was observed, indicating a metabolic shift at pH 8.2 towards ATP generation via anaerobic glycolysis (Embden-Meyerhof-Parnas pathway) (Tables 1 and 2, Figure 3). In addition, at pH 8.2, an increase in acidic end products per mg cellular protein and shift to lactate production was observed (Table 2). These changes may assist in maintenance of intracellular pH due to the lower pKa of lactic acid (3.08) compared to formic (3.75), acetic (4.75) and butanoic (4.82) acids. .

**Table 2:** **Glucose consumption and metabolic end-products produced by*F. nucleatum*grown at pH 8.2 and 7.4**

**Growth pH**	**Glucose utilisation**^**1**^	**IP**^**2**^	**Acidic end-products**^**3**^				**GDH**^**4**^
			**Lactate**	**Formate**	**Acetate**	**Butanoate**	
7.4 ± 0.1	23.1 ± 2.1	2.39 ± 0.12	5.7 ± 0.5	92.4 ± 8.6	59.4 ± 6.5	63.0 ± 5.1	8.87 ± 0.40
8.2 ± 0.1	65.9 ± 7.2	7.62 ± 0.71	18.3 ± 1.9	131.2 ± 11.6	115.3 ± 12.7	99.6 ± 10.8	13.73 ± 1.25

^1^Glucose utilisation expressed as mmoles of glucose g^-1^ cell protein.

^2^Intracellular polyglucose expressed as μg glucose mg^-1^ cell protein.

^3^Acidic end-products expressed in mmol g^-1^ cell protein.

^4^NAD-specific glutamate dehydrogenase (GDH) activity measured in cells expressed as GDH unit mg^-1^ cell protein

The proteomic results show that the concentration of six GDH isoforms was significantly altered, two isoforms showed increased concentration while four showed decreased concentration in biofilm cells (Spots 5–10, Figure 1; Table 1). Total GDH activity was investigated using enzyme assay. Biofilm cells showed a 1.5-fold increase in GDH activity compared to planktonic cells (Table 2). This finding and their reduced MW suggests that GDH isoforms (Spots 7–10, Table 1) likely represent truncated and inactive forms of the enzyme. A markedly increased (>3-fold) production of GDH compared to pH 7.4 was observed at pH 8.2 (Spots 5 and 6, Table 1). Previous proteomic results showed that when cultured at pH 7.8, *F. nucleatum* increased the production of GDH by 1.3-fold [26]. This enzyme catalyses the initial oxidation of glutamate in the 2-oxoglutarate pathway (Figure 3) and increased abundance of this enzyme would allow the organism to respond metabolically to elevated glutamate levels associated with the increased GCF flow observed in periodontal disease [51]. An increased capacity to catabolise glutamate at an elevated environmental pH may give the organism a selective advantage. Interestingly, previous studies reported differing observations with an increased intracellular concentration of GDH in an aero-tolerant strain of *F. nucleatum* subsp. *nucleatum*[39] but not in bacterial cells cultured under oxidative stress [52].

At pH 7.4, butanoate was the dominant amino acid metabolite produced by *F. nucleatum* (Table 2). This appears associated with the increased intracellular concentration of butanoate: acetoacetate CoA transferase (EC 2.8.3.9) and a decreased concentration of butyryl-CoA dehydrogenase (EC 1.3.99.2) in planktonic compared to biofilm cells (Table 1, Figure 3). Growth at pH 8.2 revealed an increased acetate/butanoate ratio (Table 2). This finding was consistent with the observed decreased expression of butyryl-CoA dehydrogenase (EC 1.3.99.2) and butanoate: acetoacetate CoA transferase (EC 2.8.3.9) and increased production of phosphate acetyltransferase (EC 2.3.1.8) in biofilm cells (Table 1, Figure 3). A shift from butanoate to acetate production by *F. nucleatum* under oxidative stress was also reported by Steeves and colleagues [52]. The production of the more oxidized end-product (acetate) yields more biomass per mole than butanoate [53]. Accordingly, it has been suggested that this shift towards acetate is energy efficient, yielding more ATP per mole of crotonoyl-CoA [54]. A decreased production of pyruvate synthase (EC 1.2.7.1) was observed in cells cultured at pH 8.2 (Table 1). This enzyme catalyses the inter-conversion of pyruvate to acetyl-CoA, linking the 2-oxoglutarate and glycolytic pathways. The decreased intracellular concentration of this enzyme potentially uncouples the two pathways in the biofilm cells (Figure 3).

### Changes in transport protein expression

Approximately 10% of bacterial genes encode for transport proteins, the majority of these are located in bacterial membranes [55]. The expression of bacterial membrane genes and proteins, particularly outer membrane proteins, is of great interest as they are directly involved in the cell’s interaction with the environment and perform essential roles in bacterial adaptation to host niches [56].

Bacterial transport proteins are classified according to their mechanism and include primary active transporters, secondary transporters, channels and pores [57]. In the present study, the intracellular concentration of 16 transport-associated proteins (five porins and 11 substrate-specific transporters) was significantly altered by a pH increase to 8.2 (Table 1). The increased intracellular concentration of TRAP transporters and increased concentration of ABC transporter binding proteins could be considered to be energy conserving as TRAP transporters rely on proton-motive force instead of ATP hydrolysis (ABC transporters) to drive the uptake of solutes from the environment. In contrast to our results, the production of TRAP transporter binding proteins was suppressed 10-fold in planktonic cells cultured at pH 7.8 [27]. The authors explained that the decreased abundance of TRAP binding proteins in planktonic cells may be due to a reduced proton gradient [27]. However, bacterial cells growing within a biofilm structure may be more protected from pH fluctuation and the loss of protons to the environment. This may explain the increased production of TRAP transporters in biofilms was observed.

The virulence of *F. nucleatum* is largely due to the adhesive properties that allow the bacterium to interact with perio-dontopathogens and host cells during the onset of periodontal disease. Two identified adhesins, RadD and FomA, are among the outer membrane proteins that are responsible for interspecies and host cell interactions [58-60]. The intracellular concentration of the adhesin FomA did not appear to be altered by planktonic *F. nucleatum* cells when cultured at pH 7.8 [27]. In the present study, however, the abundance levels of four FomA isoforms, with isoelectric points varying between 7 and 9, increased significantly in biofilm cells (Table 1). A preliminary study in the laboratory indicated that two FomA isoforms (spots 41 and 42, Figure 1 and Table 1) could be phosphorylated (data not shown) and further studies are required to determine the roles of these isofoms in biofilm cells. The protein is thought to be associated with mature plaque biofilm development as it facilitates the coaggregation between *F. nucleatum* and other bacteria such as *P. gingivalis*[60,61]. A more recent study demonstrated that in a mouse periodontitis model a bacterial suspension of *P. gingivalis* and *F. nucleatum* neutralised with anti-FomA antibody showed a significant reduction in abscess formation and gingival swelling [60]. Our results support the suggestion that FomA is a potential vaccine target for periodontal disease.

As mentioned previously, significant changes in cell morphology were associated with *F. nucleatum* biofilm formation [18]. Biofilm cells cultured at pH 8.2 presented with a significant increase in length. This altered morphology may be associated with a decrease in production of the RND superfamily antiporters (Table 1). Although better known as a multidrug-exporter, this protein also plays a role in bacterial cell division [62]. A member of the RND superfamily, EnvC protein, has been reported to be responsible for septum formation in *Escherichia coli*[63].

### Changes in stress response protein expression

**Figure 4:**
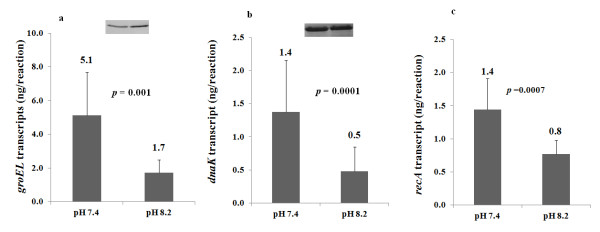
**The gene and protein expression of (a) *groEL *, (b) *recA *and (c) *dnaK *determined using either qRT-PCR or Western blotting.** Column charts represent qRT-PCR results while insets represent Western blotting results. **a**) Western blotting shows a 1.4 fold increase in GroEL protein abundance while qRT-PCR shows 3-fold decrease in *groEL* gene transcripts in biofilm cells planktonic cells. **b**) Western blotting analysis shows similar levels of RecA in both planktonic and biofilm cells while qRT-PCR shows nearly 2-fold decrease in *recA* gene expression in biofilm cells. c) qRT-PCR shows a 3-fold decrease in *dnaK* gene transcripts in biofilm cells compared to planktonic cells.

In this study, the intracellular concentrations of HSPs 70 kDa chaperone protein DnaK, 60 kDa chaperonin GroEL and peptidyl-prolyl cis-trans isomerase (PPI), and a recombination protein, RecA, were influenced by environmental pH (Table 1). Growth at pH 8.2 resulted in elevated levels of both GroEL and PPI and decrease levels of DnaK. Although constitutive, their production is influenced by stress conditions [64]. The regulation of DnaK, GroEL and PPI in response to environmental pH was also observed in previous studies [26,27]. Compared to pH 7.4, it appears that the concentration of both GroEL and PPI increase significantly at both pH 7.8 and 8.2. Our proteomic results indicate that the intracellular concentration of DnaK decreased at least 4-fold in biofilm cells (Table 1). This protein plays a role in nascent polypeptide folding and may reflect decreased growth rate and protein synthesis associated with culture at pH 8.2.Western blotting and qRT-PCR were performed to confirm the proteomic results (Figure 4). It was not possible to validate the abundance of DnaK protein using Western blotting as *F. nucleatum* DnaK failed to cross react with the mouse anti-*E. coli* DnaK monoclonal antibody used (data not shown). qRT-PCR, however, supported the proteomic results by showing a 2.9-fold decrease in expression (p < 0.01) of *dnaK* at pH 8.2 (Figure 4c). Western blotting revealed a 1.4-fold increase in GroEL (Figure 4a) while qRT-PCR gave a contrasting result indicating significantly decreased *groEL* expression (3-fold) in biofilm cells. Contrasting results were also observed in the transcript and protein levels of *recA* and its product. The proteomic data demonstrated at least 10-fold increase of RecA in biofilm cells while qRT-PCR results showed a significant 1.8-fold down-regulation of *recA* in biofilm cells (Figure 4; Table 1).

The chaperonin GroEL has been suggested to function at later stages of protein folding and is required for post-translational folding of unfolded or partially folded peptides [65] and protein degradation [66,67]. The increased intracellular concentration of this stress protein at pH 8.2 may prevent protein aggregation and misfolding due to an increased intracellular pH. Bacterial GroEL is highly homologous with human HSP 60. It was shown to cross-react with human HSP 60 on endothelial cells and induces autoimmune responses that may play a role in the process of vascular endothelial injury, a key event in the pathogenesis of atherosclerosis [68]. A recent study by Lee and colleagues [69] reported that *F. nucleatum* GroEL induces a number of risk factors in a mouse model of atheroscleorosis. The increased production of GroEL under alkaline pH environments may support the association between periodontal diseases and atherosclerosis.

The intracellular concentration of RecA, which is associated with the maintenance and repair of DNA, was found to increase at pH 8.2 (Table 1). Both acidic (<pH 5.0) and alkaline (>pH 8.0) pH environments denature DNA via depurination leading to the separation of double-stranded DNA [70,71]. Repair of the DNA gap relies on recombinational DNA proteins, including RecA [72]. The increased production of RecA may reflect the rise in intracellular pH at pH 8.2. Interestingly, our Western blotting results did not detect altered concentration of RecA in cells grown at pH 7.4 and 8.2. The production of RecA under different growth pH may therefore require further investigation although some may argue that Western blotting technique is of semi-quantitative in nature [73].

### Changes in translational protein expression

The intracellular concentration of seven proteins classified in the category of protein synthesis including five elongation factors (EF-Tu and EF-Ts) and two ribosomal S2 subunits decreased significantly by at least ten-fold at pH 8.2 (Table 1). Bacterial elongation factors EF-Tu and EF-Ts interact with each other and are essential for growth in *E. coli*[74]. These proteins are often reported to be differentially expressed by bacterial cells exposed to stressful environments. It is interesting to note that the abundance of elongation factors EF-Ts decreased 2-fold in *F. nucleatum* when exposed to pH 7.8 [26] but remained affected when the bacterium was cultured under oxidative stress [52]. Elongation factor EF-Tu has been reported to posses chaperone-like properties [75]. Len and co-workers [76] reported an increased production of EF-Tu at low pH by acid-stressed *Streptococcus mutans.* The down-regulation of EF-Tu and translational proteins in the present study may indicate reduced rate of protein synthesis at pH 8.2.

## Conclusions

To our knowledge, this is the first study to investigate alterations in both cytoplasmic and membrane protein production in *F. nucleatum* alkaline induced biofilms. Our results indicate that the biofilm cells may be more metabolically efficient, primarily via alterations in glucose and glutamate catabolism. The regulation of membrane transport proteins may assist in energy conservation. In addition, the capacity to remain functional in the suboptimal pH environment may also be attributed to the altered concentration of stress proteins. The significant increased abundance of adhesin FomA at pH 8.2 may be associated with the surface change that promotes biofilm formation. The elongation observed in bacterial cells cultured at pH 8.2 may be due to a decrease abundance of RND transporters that play a role in cells. The altered intracellular concentration three hypothetical proteins reported may be important for coping with pH stress but their roles are yet to be fully investigated. Significant changes in protein concentration were validated using a variety of techniques and generally indicated the high reliability of proteomic data. The shift to biofilm growth and the changed protein expression reflected mechanisms that likely enable *F. nucleatum* to adapt successfully and compete in its natural habitat in the oral cavity. It has been suggested that interactions between oral bacteria present in dental plaque result in many new physiological functions which cannot be observed in an individual component system [77]. Kuboniwa and colleagues (2009) examined the protein expression of *P. gingivalis* growing in a three-species system containing the pioneer plaque species *Streptococcus gordonii* and *F. nucleatum* revealing the protective mechanisms that may exist within multi-species communities [78]. The development of multi-species biofilm systems in the future may be used to increase knowledge of the gene and protein expression of *F. nucleatum*.

## Abbreviations

2DE: Two dimensional electrophoresis; GDH: Glutamate dehydrogenase; GCF: Gingival crevicular fluid; IEF: Isoelectric focusing; pI: Isoelectric point; MW: Molecular weight; PPI: Peptidyl-prolyl cis-trans isomerase; ABC: ATP binding casstte; TRAP: Tripartite ATP-indpendent periplasmic; RND: Resistance-nodulation-cell division; FomA: Fusobacterial outer membrane protein A.

## Authors’ contributions

JC conducted all hands-on experimental work and drafted the manuscript. PSZ proposed the study and provided advice on the proteomic investigation. NJG participated in the design of the experiments and provided advice on the enzyme assays and HPLC analysis. JMF provided advice and expertise from a dentist’s perspective and revised the manuscript. All authors read and approved the final manuscript.

## Supplementary Material

Additional file 1 **Table S1.** Summary of 2DE conditions used for separation of cytoplasmic and membrane proteins. Click here for file

Additional file 2 **Table S2.** Designed primers used for qRT-PCR. Click here for file

## References

[B1] Ron EZ Dworkin M, Falkow S, Rosenberg E, Schleifer KH, Stackebrandt E Bacterial stress response The prokaryotes 2006 3 Springer, New York 1012 1027

[B2] Bolstad AI Jensen HB Bakken V Taxonomy, biology, and periodontal aspects of fusobacterium nucleatum Clin Microbiol Rev 1996 9 1 55 71 8665477 10.1128/cmr.9.1.55 PMC172882

[B3] Signat B Roques C Poulet P Duffaut D Role of fusobacterium nucleatum in periodontal health and disease Curr Issues Mol Biol 2011 13 25 36 21220789

[B4] Socransky S Haffajee A Cugini M Smith C Kent R Microbial complexes in subgingival plaque J Clin Periodontol 1998 25 2 134 144 9495612 10.1111/j.1600-051x.1998.tb02419.x

[B5] Karpathy SE Qin X Gioia J Jiang H Liu Y Petrosino JF Yerrapragada S Fox GE Haake SK Weinstock GM Genome sequence of fusobacterium nucleatum subspecies polymorphum - a genetically tractable fusobacterium PLoS One 2007 2 e659 17668047 10.1371/journal.pone.0000659 PMC1924603

[B6] Cahill RJ Tan S Dougan G O'Gaora P Pickard D Kennea N Sullivan MHF Feldman RG Edwards AD Universal DNA primers amplify bacterial DNA from human fetal membranes and link fusobacterium nucleatum with prolonged preterm membrane rupture Mol Hum Reprod 2005 11 10 761 766 16254004 10.1093/molehr/gah234

[B7] Han YW Redline RW Li M Yin L Hill GB McCormick TS Fusobacterium nucleatum induces premature and term stillbirths in pregnant mice: implication of oral bacteria in preterm birth Infect Immun 2004 72 4 2272 15039352 10.1128/IAI.72.4.2272-2279.2004 PMC375172

[B8] Han YW Shen T Chung P Buhimschi IA Buhimschi CS Uncultivated bacteria as etiologic agents of intra-amniotic inflammation leading to preterm birth J Clin Microbiol 2009 47 1 38 47 18971361 10.1128/JCM.01206-08 PMC2620857

[B9] Castellarin M Warren RL Freeman JD Dreolini L Krzywinski M Strauss J Barnes R Watson P Allen-Vercoe E Moore RA Fusobacterium nucleatum infection is prevalent in human colorectal carcinoma Genome Res 2012 22 2 299 306 22009989 10.1101/gr.126516.111 PMC3266037

[B10] Kostic AD Gevers D Pedamallu CS Michaud M Duke F Earl AM Ojesina AI Jung J Bass AJ Tabernero J Genomic analysis identifies association of fusobacterium with colorectal carcinoma Genome Res 2012 22 2 292 298 22009990 10.1101/gr.126573.111 PMC3266036

[B11] Bickel M Munoz JL Giovannini P Acid–base properties of human gingival crevicular fluid J Dent Res 1985 64 10 1218 1220 3928721 10.1177/00220345850640100801

[B12] Eggert F Drewell L Bigelow J Speck J Goldner M The pH of gingival crevices and periodontal pockets in children, teenagers and adults Arch Oral Biol 1991 36 3 233 238 1908671 10.1016/0003-9969(91)90091-8

[B13] Bickel M Cimasoni G The pH of human crevicular fluid measured by a new microanalytical technique J Periodontal Res 1985 20 1 35 40 3156233 10.1111/j.1600-0765.1985.tb00408.x

[B14] Vroom JM De Grauw KJ Gerritsen HC Bradshaw DJ Marsh PD Watson GK Birmingham JJ Allison C Depth penetration and detection of pH gradients in biofilms by two-photon excitation microscopy Appl Environ Microbiol 1999 65 8 3502 3511 10427041 10.1128/aem.65.8.3502-3511.1999 PMC91526

[B15] Marsh PD Microbial ecology of dental plaque and its significance in health and disease Adv Dent Res 1994 8 2 263 271 7865085 10.1177/08959374940080022001

[B16] Takahashi N Saito K Schachtele C Yamada T Acid tolerance and acid-neutralizing activity of porphyromonas gingivalis, prevotella intermedia and fusobacterium nucleatum Oral Microbiol Immunol 1997 12 6 323 328 9573805 10.1111/j.1399-302x.1997.tb00733.x

[B17] Rogers AH Zilm PS Gully NJ Pfennig AL Marsh P Aspects of the growth and metabolism of fusobacterium nucleatum ATCC 10953 in continuous culture Oral Microbiol Immunol 1991 6 4 250 255 1812468 10.1111/j.1399-302x.1991.tb00486.x

[B18] Zilm PS Rogers AH Co-adhesion and biofilm formation by fusobacterium nucleatum in response to growth pH Anaerobe 2007 13 3–4 146 152 17540586 10.1016/j.anaerobe.2007.04.005

[B19] Takahashi N Sato T Dipeptide utilization by the periodontal pathogens porphyromonas gingivalis, prevotella intermedia, prevotella nigrescens and fusobacterium nucleatum Oral Microbiol Immunol 2002 17 1 50 54 11860556 10.1046/j.0902-0055.2001.00089.x

[B20] Shah HN Williams RAD Utilization of glucose and amino acids by bacteroides intermedius and bacteroides gingivalis Curr Microbiol 1987 15 5 241 246

[B21] Hall-Stoodley L Costerton JW Stoodley P Bacterial biofilms: from the natural environment to infectious diseases Nat Rev Microbiol 2004 2 2 95 108 15040259 10.1038/nrmicro821

[B22] Sauer K The genomics and proteomics of biofilm formation Genome Biol 2003 4 6 219 223 12801407 10.1186/gb-2003-4-6-219 PMC193612

[B23] Resch A Leicht S Saric M Pásztor L Jakob A Götz F Nordheim A Comparative proteome analysis of staphylococcus aureus biofilm and planktonic cells and correlation with transcriptome profiling Proteomics 2006 6 6 1867 1877 16470655 10.1002/pmic.200500531

[B24] Steyn B Oosthuizen MC MacDonald R Theron J Brözel VS The use of glass wool as an attachment surface for studying phenotypic changes in pseudomonas aeruginosa biofilms by two-dimensional gel electrophoresis Proteomics 2001 1 7 871 879 11503211 10.1002/1615-9861(200107)1:7<871::AID-PROT871>3.0.CO;2-2

[B25] Vilain S Cosette P Hubert M Lange C Junter G-A Jouenne T Comparative proteomic analysis of planktonic and immobilized pseudomonas aeruginosa cells: a multivariate statistical approach Anal Biochem 2004 329 1 120 130 15136174 10.1016/j.ab.2004.02.014

[B26] Zilm PS Bagley CJ Rogers AH Milne IR Gully NJ The proteomic profile of fusobacterium nucleatum is regulated by growth pH Microbiology 2007 153 1 148 159 17185543 10.1099/mic.0.2006/001040-0

[B27] Zilm PS Mira A Bagley CJ Rogers AH Effect of alkaline growth pH on the expression of cell envelope proteins in fusobacterium nucleatum Microbiology 2010 156 6 1783 1794 20299401 10.1099/mic.0.035881-0

[B28] Van der Hoeven JS De Jong MH Camp PJM Van den Kieboom CWA Competition between oral streptococcus species in the chemostat under alternating conditions of glucose limitation and excess FEMS Microbiol Lett 1985 31 6 373 379

[B29] Socransky S Manganiello A Propas D Oram V van Houte J Bacteriological studies of developing supragingival dental plaque J Periodontal Res 1977 12 2 90 106 138733 10.1111/j.1600-0765.1977.tb00112.x

[B30] Molloy MP Herbert BR Slade MB Rabilloud T Nouwens AS Williams KL Gooley AA Proteomic analysis of the escherichia coli outer membrane Eur J Biochem 2000 267 10 2871 2881 10806384 10.1046/j.1432-1327.2000.01296.x

[B31] Zhang X Shi L Shu S Wang Y Zhao K Xu N Liu S Roepstorff P An improved method of sample preparation on anchorchip™ targets for MALDI-MS and MS/MS and its application in the liver proteome project Proteomics 2007 7 14 2340 2349 17570520 10.1002/pmic.200600184

[B32] Suckau D Resemann A Schuerenberg M Hufnagel P Franzen J Holle A A novel MALDI LIFT-TOF/TOF mass spectrometer for proteomics Anal Bioanal Chem 2003 376 7 952 965 12830354 10.1007/s00216-003-2057-0

[B33] Laemmli UK Cleavage of structural proteins during the assembly of the head of bacteriophage T4 Nature 1970 227 5259 680 685 5432063 10.1038/227680a0

[B34] Irwin JA Gudmundsson HM Marteinsson VT Hreggvidsson GO Lanzetti AJ Alfredsson GA Engel PC Characterization of alanine and malate dehydrogenases from a marine psychrophile strain PA-43 Extremophiles 2001 5 3 199 211 11453464 10.1007/s007920100191

[B35] Gully NJ Rogers AH Some observations on the nutritional requirements of eikenella corrodens ATCC 23834T grown in continuous culture Oral Microbiol Immunol 1995 10 2 115 118 7675516 10.1111/j.1399-302x.1995.tb00129.x

[B36] Hamilton IR Phipps PJ Ellwood DC Effect of growth rate and glucose concentration on the biochemical properties of streptococcus mutans ingbritt in continuous culture Infect Immun 1979 26 3 861 43291 10.1128/iai.26.3.861-869.1979 PMC414699

[B37] Al-Haroni M Skaug N Bakken V Cash P Proteomic analysis of ampicillin-resistant oral fusobacterium nucleatum Oral Microbiol Immunol 2008 23 1 36 42 18173796 10.1111/j.1399-302X.2007.00387.x

[B38] da Silva VL Diniz CG dos Santos SG Gomes RMF Nicoli JR Magalhaes PP Mendes EN de Carvalho MAR Farias LM Physiological alterations of a fusobacterium nucleatum strain exposed to oxidative stress J Appl Microbiol 2006 103 1 20 26 17584449 10.1111/j.1365-2672.2006.03236.x

[B39] Silva VL Diniz CG Santos SG Carvalho MAR Farias LM Use of 2-D electrophoresis and ESI mass spectrometry techniques to characterize fusobacterium nucleatum proteins up-regulated after oxidative stress Anaerobe 2010 16 2 179 182 19683589 10.1016/j.anaerobe.2009.08.001

[B40] Görg A Drews O Lück C Weiland F Weiss W 2-DE with IPGs Electrophoresis 2009 30 S1 S122 S132 19441019 10.1002/elps.200900051

[B41] Brobey RKB Soong L Establishing a liquid-phase IEF in combination with 2-DE for the analysis of leishmania proteins Proteomics 2007 7 1 116 120 17124718 10.1002/pmic.200600587

[B42] Poetsch A Wolters D Bacterial membrane proteomics Proteomics 2008 8 19 4100 4122 18780352 10.1002/pmic.200800273

[B43] Robrish SA Thompson J Regulation of fructose metabolism and polymer synthesis by fusobacterium nucleatum ATCC 10953 J Bacteriol 1990 172 10 5714 5723 2211506 10.1128/jb.172.10.5714-5723.1990 PMC526887

[B44] Seshadri R Myers GSA Tettelin H Eisen JA Heidelberg JF Dodson RJ Davidsen TM DeBoy RT Fouts DE Haft DH Comparison of the genome of the oral pathogen treponema denticola with other spirochete genomes Proc Natl Acad Sci USA 2004 101 15 5646 5651 15064399 10.1073/pnas.0307639101 PMC397461

[B45] Gharbia SE Shah HN Welch SG The influence of peptides on the uptake of amino acids in fusobacterium; predicted interactions with porphyromonas gingivalis Curr Microbiol 1989 19 4 231 235

[B46] Robrish SA Thompson J Suppression of polyglucose degradation in fusobacterium nucleatum ATCC 10953 by amino acids FEMS Microbiol Lett 1988 55 1 29 33

[B47] Rogers AH Gully NJ Pfennig AL Zilm PS The breakdown and utilization of peptides by strains of fusobacterium nucleatum Oral Microbiol Immunol 1992 7 5 299 303 1494454 10.1111/j.1399-302x.1992.tb00592.x

[B48] Kapatral V Anderson I Ivanova N Reznik G Los T Lykidis A Bhattacharyya A Bartman A Gardner W Grechkin G Genome sequence and analysis of the oral bacterium fusobacterium nucleatum strain ATCC 25586 J Bacteriol 2002 184 7 2005 2018 11889109 10.1128/JB.184.7.2005-2018.2002 PMC134920

[B49] Rogers A Studies on fusobacteria associated with periodontal diseases Aust Dent J 1998 43 2 105 109 9612984 10.1111/j.1834-7819.1998.tb06098.x

[B50] Zilm PS Gully NJ Rogers AH Changes in growth and polyglucose synthesis in response to fructose metabolism by fusobacterium nucleatum grown in continuous culture Oral Microbiol Immunol 2003 18 4 260 262 12823803 10.1034/j.1399-302x.2003.00069.x

[B51] Syrjänen SM Alakuijala L Alakuijala P Markkanen SO Markkanen H Free amino acid levels in oral fluids of normal subjects and patients with periodontal disease Arch Oral Biol 1990 35 3 189 193 2190545 10.1016/0003-9969(90)90054-e

[B52] Steeves CH Potrykus J Barnett DA Bearne SL Oxidative stress response in the opportunistic oral pathogen fusobacterium nucleatum Proteomics 2011 11 2027 2037 21563313 10.1002/pmic.201000631

[B53] Zilm PS Gully N Rogers A Growth pH and transient increases in amino acid availability influence polyglucose synthesis by fusobacterium nucleatum grown in continuous culture FEMS Microbiol Lett 2002 215 2 203 208 12399036 10.1111/j.1574-6968.2002.tb11392.x

[B54] White R Ramezani M Gharbia S Seth R Doherty-Kirby A Shah H Stable isotope studies of glutamate catabolism in fusobacterium nucleatum Biotechnol Appl Biochem 1995 22 3 385 396 8573293

[B55] Driessen AJM Rosen BP Konings WN Diversity of transport mechanisms: common structural principles Trends Biochem Sci 2000 25 8 397 401 10916161 10.1016/s0968-0004(00)01634-0

[B56] Lin J Huang S Zhang Q Outer membrane proteins: key players for bacterial adaptation in host niches Microbes Infect 2002 4 3 325 331 11909743 10.1016/s1286-4579(02)01545-9

[B57] Gelfand MS Rodionov DA Comparative genomics and functional annotation of bacterial transporters Phys Life Rev 2008 5 1 22 49

[B58] Edwards A Grossman T Rudney J Association of a high-molecular weight arginine-binding protein of fusobacterium nucleatum ATCC 10953 with adhesion to secretory immunoglobulin A and coaggregation with streptococcus cristatus Oral Microbiol Immunol 2007 22 4 217 224 17600532 10.1111/j.1399-302X.2006.00343.x

[B59] Kaplan CW Lux R Haake SK Shi W The fusobacterium nucleatum outer membrane protein RadD Is an arginine-inhibitable adhesin required for inter-species adherence and the structured architecture of multi-species biofilm Mol Microbiol 2009 71 1 35 47 19007407 10.1111/j.1365-2958.2008.06503.x PMC2741168

[B60] Liu P-F Shi W Zhu W Smith JW Hsieh S-L Gallo RL Huang C-M Vaccination targeting surface FomA of fusobacterium nucleatum against bacterial co-aggregation: implication for treatment of periodontal infection and halitosis Vaccine 2010 28 19 3496 3505 20189489 10.1016/j.vaccine.2010.02.047 PMC2855893

[B61] Shaniztki B Hurwitz D Smorodinsky N Ganeshkumar N Weiss E Identification of a fusobacterium nucleatum PK1594 galactose-binding adhesin which mediates coaggregation with periopathogenic bacteria and hemagglutination Infect Immun 1997 65 12 5231 5237 9393820 10.1128/iai.65.12.5231-5237.1997 PMC175753

[B62] Kumar A Schweizer HP Bacterial resistance to antibiotics: active efflux and reduced uptake Adv Drug Deliv Rev 2005 57 10 1486 1513 15939505 10.1016/j.addr.2005.04.004

[B63] Saier M Tam R Reizer A Reizer J Two novel families of bacterial membrane proteins concerned with nodulation, cell division and transport Mol Microbiol 1994 11 5 841 847 8022262 10.1111/j.1365-2958.1994.tb00362.x

[B64] Feder ME Hofmann GE Heat shock-proteins, molecular chaperones, and the stress response: evolutionary and ecological physiology Annu Rev Physiol 1999 61 1 243 282 10099689 10.1146/annurev.physiol.61.1.243

[B65] Masters M Blakely G Coulson A McLennan N Yerko V Acord J Protein folding in escherichia coli: the chaperonin GroE and its substrates Res Microbiol 2009 160 4 267 277 19393741 10.1016/j.resmic.2009.04.002

[B66] Kandror O Busconi L Sherman M Goldberg AL Rapid degradation of an abnormal protein in escherichia coli involves the chaperones GroEL and GroES J Biol Chem 1994 269 38 23575 23582 7916344

[B67] Kandror O Sherman M Goldberg A Rapid degradation of an abnormal protein in escherichia coli proceeds through repeated cycles of association with GroEL J Biol Chem 1999 274 53 37743 37749 10608834 10.1074/jbc.274.53.37743

[B68] Mayr M Metzler B Kiechl S Willeit J Schett G Xu Q Wick G Endothelial cytotoxicity mediated by serum antibodies to heat shock proteins of escherichia coli and chlamydia pneumoniae : immune reactions to heat shock proteins as a possible link between infection and atherosclerosis Circulation 1999 99 12 1560 1566 10096931 10.1161/01.cir.99.12.1560

[B69] Lee HR Jun HK Kim HD Lee SH Choi BK Fusobacterium nucleatum GroEL induces risk factors of atherosclerosis in human microvascular endothelial cells and ApoE−/− mice Mol Oral Microbiol 2012 27 2 109 123 22394469 10.1111/j.2041-1014.2011.00636.x

[B70] Lindahl T Nyberg B Rate of depurination of native deoxyribonucleic acid Biochemistry 1972 11 19 3610 3618 4626532 10.1021/bi00769a018

[B71] Studier FW Sedimentation studies of the size and shape of DNA J Mol Biol 1965 11 2 373 390 14290352 10.1016/s0022-2836(65)80064-x

[B72] Webb BL Cox MM Inman RB Recombinational DNA repair: the RecF and RecR proteins limit the extension of RecA filaments beyond single-strand DNA gaps Cell 1997 91 3 347 356 9363943 10.1016/s0092-8674(00)80418-3

[B73] Aldridge GM Podrebarac DM Greenough WT Weiler IJ The use of total protein stains as loading controls: an alternative to high-abundance single-protein controls in semi-quantitative immunoblotting J Neurosci Methods 2008 172 2 250 254 18571732 10.1016/j.jneumeth.2008.05.00 PMC2567873

[B74] Alexander C Bilgin N Lindschau C Mesters JR Kraal B Hilgenfeld R Erdmann VA Lippmann C Phosphorylation of elongation factor Tu prevents ternary complex formation J Biol Chem 1995 270 24 14541 14547 7782317 10.1074/jbc.270.24.14541

[B75] Caldas TD Yaagoubi AE Richarme G Chaperone properties of bacterial elongation factor EF-Tu J Biol Chem 1998 273 19 11478 9565560 10.1074/jbc.273.19.11478

[B76] Len ACL Harty DWS Jacques NA Stress-responsive proteins are upregulated in streptococcus mutans during acid tolerance Microbiology 2004 150 5 1339 1351 15133096 10.1099/mic.0.27008-0

[B77] Kuramitsu HK He X Lux R Anderson MH Shi W Interspecies interactions within oral microbial communities Microbiol Mol Biol Rev 2007 71 4 653 670 18063722 10.1128/MMBR.00024-07 PMC2168648

[B78] Kuboniwa M Hendrickson EL Xia Q Wang T Xie H Hackett M Lamont RJ Proteomics of porphyromonas gingivalis within a model oral microbial community BMC Microbiol 2009 9 1 98 112 19454014 10.1186/1471-2180-9-98 PMC2689231

