# Supplementary material for: A proteomic investigation of Fusobacterium nucleatum alkaline-induced biofilms
Source: BMC Microbiol. 2012 Sep 3;12:189. doi: 10.1186/1471-2180-12-189 (PMC3478200; doi:10.1186/1471-2180-12-189)
Supplement: Additional file 1 — Table S1. Summary of 2DE conditions used for separation of cytoplasmic and membrane proteins. [file 1471-2180-12-189-S1.doc]

**Additional File 1**: Summary of 2DE conditions used for separation of cytoplasmic and membrane proteins.

| **Protein preparation** | **pI range** | **IPG strip length (cm)** | **Protein load (µg)** | **IEF conditions** | **Protein stain** |
| --- | --- | --- | --- | --- | --- |
| MP^1^ | 4-7 | 11 | 250 | 150 V – 1 h  300 V – 2 h  3000 V – 2 h  8000 V– 5 h  30,000 Vh  500 V– 10 h | Coomassie Blue R-250* |
|  | 7-10 | 11 | 400 | 150 V – 1 h  300 V – 2 h  3000 V – 2 h  8000V – 5 h  30,000 Vh  500 V – 10 h | Coomassie Blue R-250* |
| CP^2^ | 4-7 | 17 | 1200 | 150 V – 1 h  300 V – 2 h  3000 V – 2 h  10,000 V – 5 h  50,000 Vh  500 V – 10 h | Coomassie Blue R-250* |
|  | 7-10 | 11 | 100 | 150 V – 1 h  300 V – 2 h  3000 V – 2 h  8000 V – 5 h  20,000 Vh  500 V – 10 h | Flamingo Fluorescent Stain (Bio-  Rad)^ |

^1^MP – membrane proteins

^2^ CP – cytoplasmic proteins

*Gels were scanned using a GS-800 Densitometer (Bio-Rad Laboratories, CA, USA)

^Gels were scanned using a Typhoon Scanner (GE Healthcare, Buckinghamshire, UK)
