# Supplementary material for: A proteomic investigation of Fusobacterium nucleatum alkaline-induced biofilms
Source: BMC Microbiol. 2012 Sep 3;12:189. doi: 10.1186/1471-2180-12-189 (PMC3478200; doi:10.1186/1471-2180-12-189)
Supplement: Additional file 2 — Table S2. Designed primers used for qRT-PCR. [file 1471-2180-12-189-S2.doc]

**Additional File 2**: Designed primers used for qRT-PCR

| **Gene** | **Primer sequence** | | **Primer length (bp)** | **GC%** | **T_m_ (ºC)** | **Product size (bp)** |
| --- | --- | --- | --- | --- | --- | --- |
| *dnaK* | F (5’-3’) | GTATCCCTGCTGCTCCAA | 18 | 56 | 54 | 184 |
|  | R(3’-5’) | GTGCTTCTGCTTCCTTAGTC | 20 | 50 | 52 |  |
| *groEL* | F (5’-3’) | ATTGACCCAGCAAAAGTTAC | 20 | 40 | 52 | 137 |
|  | R(3’-5’) | GGCATCATTCCACCAGCA | 18 | 56 | 58 |  |
| *recA* | F (5’-3’) | TGGCAGCGATTACAAAAGG | 19 | 47 | 57 | 269 |
|  | R(3’-5’) | CATAAACTGGGTCAAGAGCAT | 21 | 43 | 55 |  |
| 16 S rRNA | R(3’-5’) | GCTCGTGTCGTGAGATGTT | 19 | 53 | 53 | 153 |
|  | R(3’-5’) | CCAGCGTATAAGGGGCA | 17 | 59 | 55 |  |

F: forward primer; R: reverse primer
